# Does whole body vibration exercise improve oxidative stress markers in women with fibromyalgia?

**DOI:** 10.1590/1414-431X20198688

**Published:** 2019-08-05

**Authors:** J.M. Santos, V.A. Mendonça, V.G.C. Ribeiro, R. Tossige-Gomes, S.F. Fonseca, A.C.N. Prates, J. Flor, A.C.C. Oliveira, J.B. Martins, B.C.C. Garcia, H.R. Leite, P.H.S. Figueiredo, M. Bernardo-Filho, A.C.R. Lacerda

**Affiliations:** 1 Programa de Pós-Graduação em Reabilitação e Desempenho Funcional, Universidade Federal dos Vales do Jequitinhonha e Mucuri, Diamantina, MG, Brasil; 2 Departamento de Fisioterapia, Universidade Federal dos Vales do Jequitinhonha e Mucuri, Diamantina, MG, Brasil; 3 Programa Multicêntrico de Pós-graduação em Ciências Fisiológicas, Sociedade Brasileira de Fisiologia, Diamantina, MG, Brasil; 4 Departamento de Biofísica e Biometria, Instituto de Biologia Roberto Alcântara Gomes, Universidade do Estado do Rio de Janeiro, Rio de Janeiro, RJ, Brasil

**Keywords:** Whole body vibration, Chronic disease, Oxidative stress, Fibromyalgia

## Abstract

The objective of this study was to investigate the effect of whole body vibration (WBV) exercise on oxidative stress markers in a group of women with fibromyalgia (FM) compared to a group of healthy women (CT). Twenty-one women diagnosed with FM and 21 age- and weight-matched healthy women were enrolled the study. Plasma oxidative stress markers (primary outcomes) were evaluated at rest and after WBV, and included thiobarbituric acid reactive substances (TBARS), iron reduction capacity (FRAP), superoxide dismutase antioxidant enzymes activity (SOD), and catalase (CAT). At rest, the FM group had higher TBARS (P<0.001) and FRAP (P<0.001), and lower CAT (P=0.005) compared to the CT. In the CT group, the WBV had no effect on TBARS (P=0.559) and FRAP (P=0.926), whereas it increased both SOD (P<0.001) and CAT (P<0.001). In the FM group, the WBV reduced TBARS (p <0.001), FRAP (P<0.001), and CAT (P=0.005), while it increased SOD (P=0.019). There was an interaction effect (moments *vs* groups) in the TBARS (effect size=1.34), FRAP (effect size=0.93), CAT (effect size=1.45), and SOD (effect size=1.44) (P<0.001). A single trial of WBV exercise improved all oxidant and antioxidant parameters towards a greater adaptation to the stress response in FM women.

## Introduction

Fibromyalgia (FM) syndrome is a chronic disorder characterized by generalized and persistent musculoskeletal pain that predominantly affects women (between 61 and 90% of affected persons). The estimated prevalence is of 2 to 4% in the general population. Numerous symptoms may also be associated with this syndrome, including pain points, fatigue, anxiety, depression, lack of restful sleep, muscle stiffness, or irritable bowel syndrome (1,2).

Oxidative stress is a relevant event in the pathogenesis of FM, resulting in an imbalance between oxidative and antioxidant factors, favoring marked production of reactive oxygen species (ROS) and reactive nitrogen species (RNS) (3). RNS are highly reactive chemical species with an unmatched electron formed by catalyzing transition metals like iron, copper, or manganese. These toxic molecules become highly reactive in their formation because of their altered number of unmatched valence electrons. These RNS are suggested to play important roles in rheumatologic conditions like rheumatoid arthritis, ankylosing spondylitis, and chronic fatigue syndrome (CFS) (4,5). There is little information on oxidative stress in FM, but the level of malondialdehyde is higher and superoxide dismutase is lower than that of the control (6,7). In addition, studies report an association between oxidative stress and insulin resistance suggesting that increased levels of ROS are an important triggering factor for insulin resistance in various contexts (8). Fava et al. (9) reported that substrate changes of the insulin receptor are sensitive to changes in cognitive status in FM.

The management of FM is based on symptomatic multidisciplinary treatment through pharmacologic and non-pharmacologic strategies. Among non-pharmacologic treatments, exercise, cognitive behavioral therapy, and education have the strongest efficacy evidence (10,11). Bote and colleagues demonstrated that mild cycling improves the inflammatory and stress status of FM. However, exhaustive exercise cannot be considered an anti-inflammatory stimulus (12,13).

Whole body vibration (WBV) may represent an alternative strategy for the treatment of symptoms associated with FM (14,15) and other chronic inflammatory diseases (16–18). The vibratory platform generates a mechanical stimulus that increases the muscles gravitational load (14), producing a defensive response from the muscular system to increase stiffness and reduce vibration transmission in the body. This mechanism results in the activation of the tonic vibration reflex, producing a reflex muscle contraction (14).

A recent study by our team showed that an exercise trial of WBV stimulus, considered of mild and short effort, seems to be sufficient to cause a modulation in inflammatory biomarkers, in the sense of adjustment of homeostasis of the inflammation in FM subjects compared to a control group (15). However, as far as we know, no study investigated whether WBV exercise could improve the oxidative stress markers in FM subjects. Thus, the aim of the current study was to investigate effects of a single WBV exercise on oxidative stress markers in FM women and healthy matched women.

## Material and Methods

### Ethical statement

This investigation was conducted in accordance with the ethical principles for research involving humans (principles of the Declaration of Helsinki) and Brazilian Guidelines (Res. CNS 196/96, No. RBR-36d8nf), received approval from the Ethics Committee of the Universidade Federal dos Vales do Jequitinhonha e Mucuri (No. 2.022.894), and was submitted to the Registry of Clinical Trials (REBEC) (RBR-83KYSP). Subjects were recruited through the waiting list of the clinical school of physiotherapy at the Universidade Federal dos Vales do Jequitinhonha e Mucuri and from advertisements in the Basic Health Units and on the radio station Diamantina, Brazil. All subjects were informed about the study procedures and provided their written consent to participate in this study.

### Study design

This was an experimental matched case-control study that assessed variables before and immediately after a single trial of WBV exercise. The sample size was calculated from a pilot study consisting of 8 subjects diagnosed with fibromyalgia and 8 healthy matched subjects, in which we evaluated the effect of WBV exercise on plasma TBARS level before and after a single WBV exercise intervention. Thus, a sample size of 42 subjects (21 FMs and 21 controls) was required for an effect size of 0.25, power of 0.80, significance level of 0.05, using two-way ANOVA (main effects and interactions).

### Study population

Inclusion criteria were diagnosis as FM according to the American College of Rheumatology revised preliminary diagnostic criteria of fibromyalgia (1,2), female gender, and acceptance to participate in the study. The exclusion criteria were the presence of any concomitant disease that could be exacerbated by physical activity, pregnancy, inflammatory diseases, degenerative, joint, respiratory, or cardiovascular disease, treatment by a psychiatrist, or those who performed physical activity more than twice a week. Individuals who presented some of the possible contraindications for the WBV stimulus, such as acute hernia, stroke, diabetes, epilepsy, metabolic or neuromuscular diseases, and orthopedic and prosthetic lesions were also excluded.

**Figure 1:**
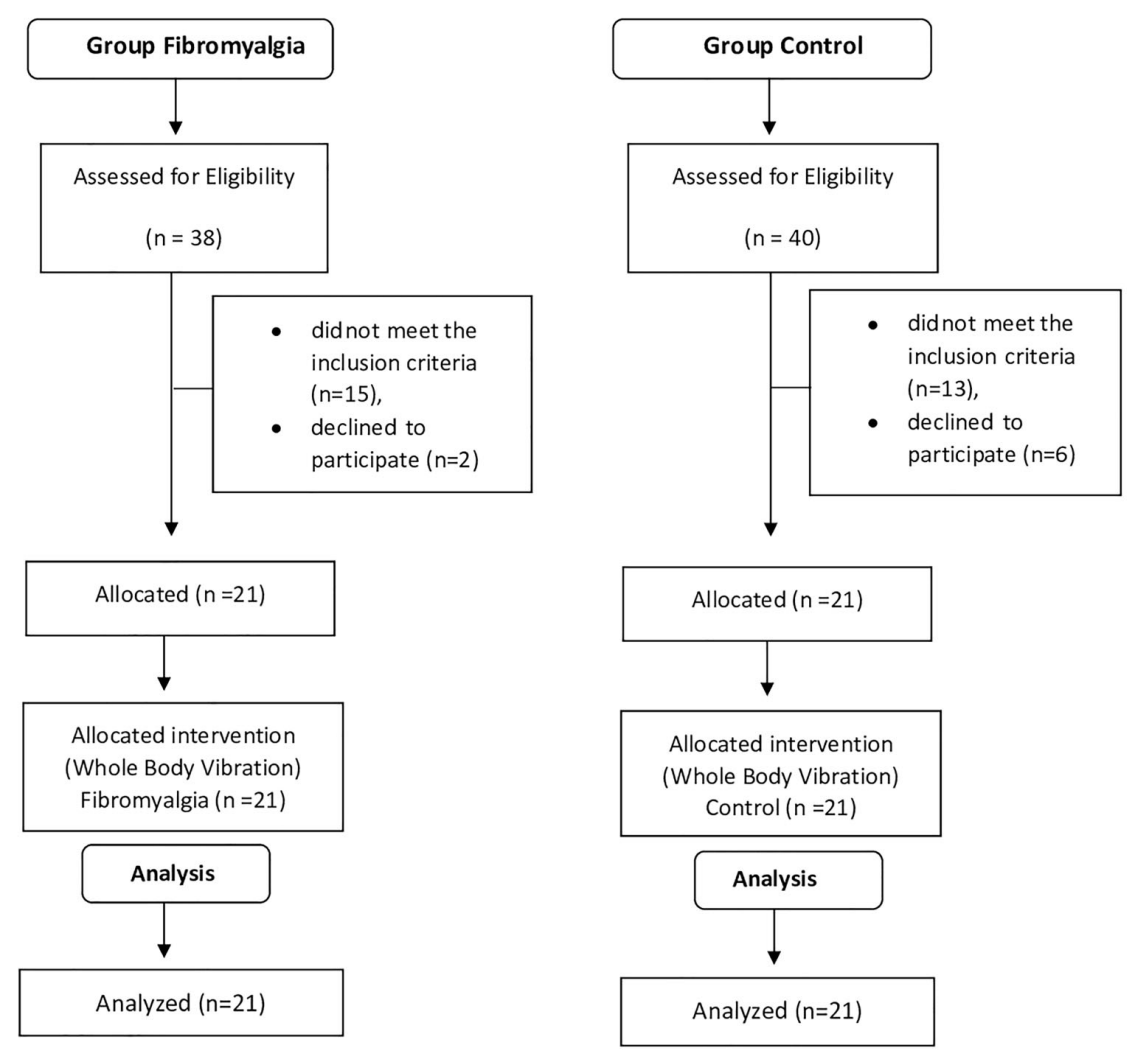
Flowchart of the study.

A total of 38 women diagnosed with FM were initially screened for eligibility. Of these, fifteen did not meet the inclusion criteria (n=15) and two refused to participate (n=2). The remaining 21 women were allocated to the FM group. A total of 40 asymptomatic women were initially screened for eligibility. Of these, thirteen were excluded because they did not meet the inclusion criteria (n=13) and six refused to participate (n=6). The remaining 21 women were allocated to the control group. Thus, 21 female FM subjects and 21 age- and weight-matched healthy women were enrolled the study (Figure 1).

The groups were matched by age, body mass, height, and medication intake. The same procedures were applied to both groups: complete a questionnaire about their lifestyle (diet, habits, etc.), medication, and other previous or current concomitant diseases, anthropometric characterization, evaluation of tender points, questionnaires, and muscular performance test of the lower limbs. The subjects participated independently of the experimental situations and the evaluation was conducted by the same investigator to ensure similar instruction. Analysis of questionnaires was performed by a blind researcher.

### Clinical assessments

After 8 hours of fasting, a blood sample was collected for analysis of the insulin resistance by the Homa-ir index (fasting glucose × fasting insulin / 22.5 (reference values: ≤3.40)) and Homa-beta index ((20 × fasting insulin) / (fasting glycemia) – 3.5 (reference values: 167.0–175.0)) (19).

The next day, participants were submitted to a preliminary section for familiarization with the experimental procedures, application of self-reported instruments, evaluation of pain points, physical test, and anthropometric measures. Body weight was measured by an anthropometric mechanical scale, equipped with a stadiometer (Seca, Germany). The body mass index (BMI) was determined as the total body weight divided by height squared (kg/m^2^) (20). The assessment of tender points was performed by palpation. A tender point was considered positive when painful discomfort was triggered after digit pressure of around 4 kg/cm (1). The health status, functional capacity, and main symptoms of FM were assessed using the Brazilian version of the Fibromyalgia Impact Questionnaire (FIQ) (21). Screening of depression was performed using the Beck Depression Inventory (BDI) (22). Muscle strength of the lower limbs was evaluated by the sit-to-stand-up test (SUT) (23).

One week after the preliminary section, always in the morning, all subjects performed the experimental situation. Blood samples were collected before and immediately after WBV exercise for analysis of oxidative stress markers (primary outcomes). Moreover, before and immediately after the WBV exercise, all subjects had the Subjective Perception of Effort (secondary outcome) evaluated using a numerical scale of 6 to 20 (Borg scale), where the individual pointed out his own perception of effort at that moment.

### Intervention

All subjects arrived at the experimental center at 7:30 am, after fasting for at least 8 h, including not taking regular medication. The experimental protocol began with the subjects remaining at rest for 30 min. During this period, the subjects were instructed to remain seated and not to perform sudden movements. The vibration exposure consisted of performing squatting exercises with a vibration stimulus (frequency of 40 Hz and amplitude of 4 mm) performed on a commercial model of a vibration platform (FitVibe, GymnaUniphy NV, Belgium). This vibration frequency and amplitude was selected because this prototype renders an acceleration range of 2–5 g (14). Acceleration is a variable used to quantify the intensity of the vibration stimulus (14).

During vibration stimulus, the subjects performed 8 series of 40 s of squatting exercises. In each exercise series, the volunteer was instructed, by the same examiner blind to the group assignments of the subjects, to perform a semi-complete knee extension (angle 10°) to knee bend (angle 60°). The 60° angle was measured for each volunteer by using a universal goniometer before initiating the exercise series, and a barrier was placed at the gluteal region to limit the flexion degree of the knees.

To control the time of each squat, an examiner instructed the individuals to bend their knees to a 60° angle for 3 s and then to an 10° angle for 3 s, over the 40 s of each series, for a total of 5 repetitions each. The subjects were also instructed to remain in the correct position with their feet on the platform and their spine, arms, and head also in the instructed position (24).

### Determination of oxidative stress markers

Blood samples were collected aseptically by puncture of the median cubital vein. The tubes containing EDTA were centrifuged at 3000 *g* for 10 min at 20^o^C to remove cells and debris and were stored as plasma and erythrocytes aliquots at –80°C. Oxidative stress markers were evaluated by determining plasma levels of lipid peroxidation products (thiobarbituric acid reactive substances (TBARS)) (25), enzymatic antioxidants (erythrocyte activity of the enzymes superoxide dismutase (SOD) and catalase (CAT)) (26,27), and non-enzymatic antioxidants total antioxidant capacity of plasma (FRAP) (28) according to previously published methods. TBARS level is reported in nanomoles MDA per milligram of protein, SOD activity is reported in units (U) per milligram of protein, and CAT activity is reported by ΔE/min per milligram protein, where ΔE represents the variation in enzyme activity for 1 min. The total antioxidant capacity is reported as micrograms FeSO_4_ per milligram of protein.

### Statistical analyses

The data were analyzed using the statistical package, SPSS version 22.0 (IBM, USA), Graph-Pad Prism version 5.0 (GraphPad Software, USA), and Sigma Stat (Systat Software, USA). The data are reported as means±SE. The Shapiro-Wilk and Levene tests were applied to evaluate the normality and homogeneity of the results, respectively. The Student's unmatched *t*-test or Mann Whitney test were used to compare measurements between the groups at baseline. The effects of WBV exercise were evaluated by two-way ANOVA mixed test, which compares the main effects in relation to time (pre and post) and the interaction between time (pre and post) and groups (control × intervention). The *t*-test was used for *post hoc* comparisons. The statistical significance level was set at P≤0.05. All efficacy analyses were based on the intent-to-treat population.

## Results

There was no difference between the FM and CT groups with regard to age, body mass, height, or BMI (P>0.05). The FM group had a greater number of tender points than the CT group (P<0.0001). The FM group had higher scores on BDI (46%), where scores of 17–27 were classified as mild depression. The minimum time from diagnosis in the FM group was two years. A score of 69.76±3.12 was found in the FM group for the FIQ. The CT group showed a higher number of repetitions (15.92±2.55) compared to the FM group (7.96±0.55) during SUT, characterizing reduced muscle performance of lower limbs in the FM group. Homa-ir index was present in 76% of individuals with FM and the Homa-beta index was elevated in 67% of these individuals, of whom 14% were newly diagnosed with diabetes mellitus (Table 1).

**Table 1:** Characteristics of study subjects.

	CT (n=21)	FM (n=21)	P value
Age (years)	50.09±10.12	52.04±8.18	0.82
Weight (kg)	69.80±8.85	70.16±9.55	0.94
Height (m)	1.60±0.06	1.59±0.05	0.58
BMI (kg/m^2^)	26.81±3.21	27.79±3.40	0.82
Tender points	0.95±0.55	16.1±1.18*	<0.0001
Time from diagnosis (years)	–	>2	–
BDI (score)	9.16±0.55	22.96±1.55*	0.0002
SUT (number)	15.92±2.55	7.96±0.55*	<0.0001
FIQ (score)		69.76±3.12	
HOMA IR (index)	1.94±0.80	3.87±1.43*	<0.0001
HOMA BETA (index)	119.71±35.22	238.86±108.89*	<0.0001
TBARS (nmol MDA/mg prot)	0.19±0.01	0.78±0.26*	<0.0001
FRAP (nmol FeSO_4_/mg prot)	188.26±17.17	485.57±45.54*	<0.0001
CAT (U/mg prot)	7.49±3.92	3.83±1.91*	0.005
SOD (U/mg prot)	1.08±0.43	0.93±0.15	0.16

CT: healthy control; FM: fibromyalgia; BMI: body mass index; BDI: Beck Depression Inventory; FIQ: Fibromyalgia Impact Questionnaire; SUT: Sit-to-stand-up test; Homa IR: resistance to insulin; TBARS: thiobarbituric acid reactive substances; FRAP: plasma iron reduction capacity; CAT: catalase; SOD: superoxide dismutase. Data are reported as means±SE. *P≤0.05 Student's unpaired *t*-test (BDI, SUT, FIQ) and Mann Whitney (Homa ir, Homa beta).

At rest, the FM group had higher plasma level of TBARS and FRAP, and a lower plasma level of CAT, with no difference of the plasma level of SOD compared to the CT group.

In the CT group, the WBV exercise trial had no effect on the plasma level of TBARS and FRAP, whereas the stimulus increased the plasma level of both SOD and CAT. In the FM group, the WBV exercise trial promoted a reduction in the plasma level of TBARS, FRAP, and CAT, and increased the plasma level of SOD.

**Figure 2:**
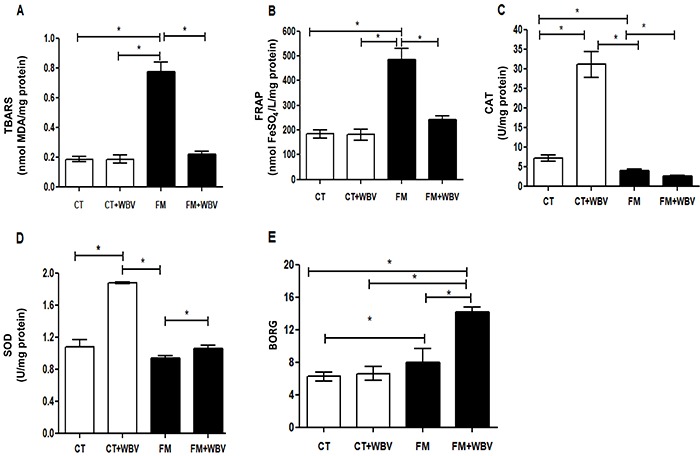
Treatment effects for oxidative markers in healthy controls (CT) and fibromyalgia (FM) female patients treated or not with whole body vibration (WBV) exercise. **A**, thiobarbituric acid reactive substances (TBARS); **B**, iron reduction capacity (FRAP); **C**, catalase (CAT); **D**, superoxide dismutase antioxidant enzymes activity (SOD); **E**, Borg scale scores. Data are reported as means±SE. *P<0.05 (ANOVA).

There was an interaction effect in the plasma levels of TBARS (effect size=1.34), FRAP (effect size=0.93), CAT (effect size=1.45), and SOD (effect size=1.44), and in the Borg scale (effect size=3.03) (P<0.001). Moreover, the Borg scale differed between groups at rest (P<0.0001) and the WBV exercise trial increased the subjective effort perception solely in the FM group (P<0.0001), while in the control group the subjective effort perception remained unchanged (P=0.091). The subjective effort perception was classified as mild according to the American College of Sports Medicine (29) in both groups (Figure 2).

## Discussion

To the best of our knowledge, this is the first study investigating the effects of a single trial of WBV exercise on oxidative stress markers in FM women compared to a healthy matched group. The findings of this study demonstrated that FM subjects exhibited an oxidative stress profile compared to control, and the WBV exercise trial modulated oxidant and antioxidant parameters in female FM subjects.

It has been proposed that oxidative stress may play a role in the etiopathogenesis of FM (30). Thus, Bagis and colleagues used malondialdehyde (MDA) level (a toxic metabolite of lipid peroxidation) as a possible indicator of an increase in free radicals in FM subjects, and found a significant increase compared to the control group (31). Likewise, Ozgocmen and colleagues reported higher TBARS plasma levels in FM subjects compared to the control group (7).

Regarding the total antioxidant capacity, FM subjects showed higher plasma levels compared with control group at baseline. These results seemed paradoxical at first because two studies found a low total antioxidant status in FM subjects (7,8). Ozgocmen and colleagues investigated SOD activities for evaluation of antioxidant status and found no significant difference between the FM and control groups (15). However, Bagis and colleagues reported that blood SOD activity was significantly lower in FM subjects than in the control group (31). Similarly, Sendur and colleagues showed that the blood CAT activity in FM subjects was significantly lower than in the control group (32). Finally, one study that evaluated the blood thyroid hormones to investigate the antioxidant capacity in FM subjects compared to the control found a lower level in FM subjects (33). Our findings demonstrated the occurrence of increased oxidative stress and imbalance between oxidant and antioxidant status in FM subjects compared to the control group.

Current evidence points to the presence of mitochondrial dysfunction and the consequent increase of ROS especially in muscle and in peripheral and central nervous tissue, as a plausible hypothesis that potentially explains pain and fatigue in CFS and FM subjects. These oxygen radicals are produced by the mitochondria. The increased number of free radicals in the mitochondria is a consequence of a negative balance between ROS production and antioxidant defense. Thus, the relative rates of ROS production and decomposition determine their steady-state level and the potential to cause tissue injury. The production of ROS increases due to an increased frequency of action potentials registered in the neurons of the pain system (peripheral and central) due to a traumatic injury or infection that often precedes the syndromes. In this way, different cells will become necrotic and several humoral factors, such as bradykinin and potassium, will leak into the environment, possibly targeting nociceptors of the pain neurons. In this case, a constant imbalance at the level of the membrane potential occurs, which has to be restored by Na/K-ATPase pumps. These pumps are ATP-dependent, which stresses the mitochondrial function, resulting in an increased production of oxygen radicals. This hypothesis is supported by Meeus et al. and Sánchez-Domínguez et al. who reported that mitochondria in axons and presynaptic terminals provide sources of ATP to drive the ion pumps concentrated in these structures for rapid restoration of ion gradients following depolarization and neurotransmitter release (34,35). Moreover, there is evidence demonstrating that changed oxidative stress parameters are associated with severity of FM, including physical symptoms (pain and fatigue) and worse functional performance (36).

Recent hypotheses of FM etiology have highlighted inflammatory disorders accompanied by changes in redox imbalance (5,26). Studies have shown that subjects with FM show increased plasma levels of TBARS compared to healthy individuals (31). The only baseline study that addressed exercise strategies in parameters of oxidative stress in FM observed significant decreases in oxidative stress parameters and improvement of clinical data in FM subjects following the 12-week combined exercise program, thus indicating that exercise therapy can significantly reduce the oxidative load. In this study, women with FM presented higher plasma level of TBARS compared to resting healthy individuals, characterized by the presence of redox imbalance, which may lead to an inflammatory profile in the FM subjects (37).

The literature points out that balanced oxidative stress and chronic systemic low-grade inflammation are potent mediators of homeostasis (5). Exercise seems to have the capacity to transiently provoke a response in both biological systems (26). While excessive amounts of ROS are detrimental, transient exercise-induced changes are now recognized as integral agents in promoting adaptation (38). Patient education, cognitive behavioral therapy, pharmacotherapy, good sleeping patterns, and combined exercise are recommended for the treatment for FM. Specifically, exercise has favorable effects on physical fitness, aerobic performance, pain management, and quality of life of FM subjects (39). However, the effect of WBV exercise on the balance of oxidative stress parameters in this population is unknown, especially compared to healthy matched control subjects.

In the current study, the intensity of the WBV exercise was classified as mild in both groups, confirming previous data of our group (15). Moreover, the FM group exhibited greater subjective effort perception at rest and a further increase after the WBV exercise, whereas the subjective effort perception remained unchanged in the control group. Moreover, FM subjects had lower physical performance and attenuated physical and emotional scores in the FIQ, supporting that they changed their physical effort perception. The FM group also presented abnormalities in glucose metabolism compared to the control group. Thus, insulin resistance was present in 76% of FM subjects, of whom 14% were newly diagnosed with diabetes mellitus. Researchers have already identified the occurrence of insulin resistance in subjects with FM, which can lead to deficits and cognitive abnormalities (9). Authors suggest that exercise-induced oxidative stress ameliorates insulin resistance and causes an adaptive response promoting endogenous antioxidant defense capacity (40).

Physical exercise has many benefits, but it can also have a negative impact on the body, depending on the intensity and volume of physical exertion, sex, age, and fitness level. The negative effects of physical exercise are commonly attributed to an imbalance between levels of antioxidants (both low molecular weight antioxidants and antioxidant enzymes) and reactive oxygen and nitrogen species, due to the excessive production of free radicals during exercise. Consistent with the literature and given the importance of appropriate exercise strategies to maintain redox balance, we can consider that a single trial of WBV exercise was beneficial for both FM and healthy matched individuals.

The antioxidant enzymes SOD, CAT, and glutathione peroxidase (GPX) are the primary defense against ROS generated during physical exercise, and they increase in response to physical exercise. The deleterious effects of redox imbalance seem to occur particularly after non-regular intense exercise, while regular training has positive effects, influencing cellular processes that lead to increased expression of antioxidants, which then provide better protection against ROS during physical exercise training. Therefore, we suppose that our WBV exercise trial can be considered beneficial for the modulation of the redox balance as it is classified as a mild intensity stimulus (15).

Our data suggest that a single trial of WBV exercise was sufficient to increase antioxidants by promoting additional protection against oxidative stress in both groups. However, it is important to note the significant differences in CAT response to the WBV stimulus. This demonstrates either that FM may be associated with decreased antioxidant defense ability or that only a single WBV exercise was not able to activate these defensive pathways in FM subjects, whereas in the control group the WBV stimulus tended to increase all the endogenous antioxidant capacities parameters (CAT and SOD). The reduction in the total antioxidant capacity (FRAP) in the FM group by the WBV exercise reaching values close to those of healthy matched controls at their basal status seemed paradoxical at first. However, this did not involve prejudice related to cellular damage, since we observed an increase in the SOD enzyme activity. Thus, we suppose that this augment in the SOD enzyme activity could have reduced TBARS level, in the direction of reducing oxidative damage in subjects with FM.

A particular strength of the present study was that it was performed under controlled and standardized conditions. However, since this is a case-control study, with subjects matched 1:1, the inter-group comparisons should be interpreted with caution. In addition, as this investigation was only performed with women, caution should be taken to generalize the conclusions. Moreover, it is not possible to assume the isolated effect of WBV since methodologically the stimulus will always be associated with muscle contraction due to the squat position. Thus, WBV exercise was associated with knee flexion and muscle contraction of the lower limb (squat position) to avoid the phenomenon of resonant catastrophe, i.e., the tendency of a mechanical system (human body) to respond at greater amplitude when the frequency of its oscillations matches the system's natural frequency of vibration (vibratory platform) than it does at other frequencies. As the resonance frequency increases with the lower limbs in extension, a squat position helps to avoid the resonant catastrophe once the transmissibility of the vibration will depend on the musculoskeletal stiffness. Finally, because the literature on oxidative stress often focuses on acute analysis, i.e., effect of an exercise intervention trial, a gap remains regarding sustained protection due to adaptations to subsequent oxidative stressors, especially those that are not induced by a WBV exercise, i.e., endogenous antioxidants.

We demonstrated that a mild single trial of WBV exercise modulated all parameters of oxidative stress and antioxidant defense towards a greater adaptation to the stress response in FM women. Further analysis involving double blind placebo-controlled clinical trials will be required to analyze the effect of WBV training on oxidative stress markers in FM. Our research group is currently working in this direction, on the basis of the conclusions of the exploratory work discussed in this article.

The results described in this article may help design experiments to better understand the influence of the sum of several WBV exercises in the modulation of oxidative stress, which is an important event in the pathogenesis of FM.
